# Common and uncommon neuroimaging manifestations of ataxia: an illustrated guide for the trainee radiologist. Part 1 - acquired diseases

**DOI:** 10.1590/0100-3984.2021.0111

**Published:** 2022

**Authors:** Vinicius de Menezes Jarry, Fernanda Veloso Pereira, Mariana Dalaqua, Juliana Ávila Duarte, Marcondes Cavalcanti França Junior, Fabiano Reis

**Affiliations:** 1 Department of Radiology, Universidade Estadual de Campinas (Unicamp), Campinas, SP, Brazil.; 2 Hôpitaux Universitaires de Genève, Service de Radiologie, Geneva, Switzerland.; 3 Department of Radiology and Diagnostic Imaging, Hospital de Clínicas de Porto Alegre (HCPA), Porto Alegre, RS, Brazil.; 4 Department of Neurology, Universidade Estadual de Campinas (Unicamp), Campinas, SP, Brazil.

**Keywords:** Neuroimaging, Cerebellar ataxia, Cerebellar nuclei, Magnetic resonance imaging, Neuroimagem, Ataxia cerebelar, Núcleos cerebelares, Ressonância magnética

## Abstract

Ataxia is defined as a lack of coordination of voluntary movement, caused by a variety of factors. Ataxia can be classified by the age at onset and type (chronic or acute). The causative lesions involve the cerebellum and cerebellar connections. The correct, appropriate use of neuroimaging, particularly magnetic resonance imaging, can make the diagnosis relatively accurate and facilitate implementation of the appropriate clinical management. The purpose of this pictorial essay is to describe the imaging findings of ataxia, based on cases obtained from the archives of a tertiary care hospital, with a review of the most important findings. We also review and discuss the imaging aspects of infectious, toxic, vascular, and inflammatory diseases.

## Abstract (pt)

Ataxia é definida como uma síndrome de falta de coordenação dos músculos de movimentação voluntária. Vários fatores podem causar ataxias, os quais podem ser classificados de acordo com a idade, tipo de evolução (crônica ou aguda), cujas lesões envolvem o cerebelo e as conexões cerebelares. Com o uso correto e apropriado da neuroimagem, particularmente da ressonância magnética, o diagnóstico pode ser relativamente preciso e o manejo clínico pode ser implementado de maneira correta. O objetivo deste artigo é descrever os achados de imagem na síndrome atáxica com base em casos recuperados do arquivo digital de um hospital terciário, com a revisão dos principais achados de imagem. Neste ensaio revisamos e discutimos os aspectos imagem de doenças infecciosas, tóxicas, vasculares e inflamatórias.

## INTRODUCTION

Ataxia is defined as a lack of coordination of voluntary muscle movement, caused by a variety of factors. Its manifestations include gait ataxia, dysarthria, nystagmus, sensory and truncal ataxia, dysdiadochokinesia, intention tremor, dysmetria, and eye movement disorders^(1)^. Ataxia can be classified on the basis of various aspects^(2)^: chronology, clinical course (acute or chronic), distribution (focal or generalized), and type (hereditary or acquired). The age at onset may suggest a congenital or developmental etiology, including genetic causes that manifest even in young adults^(2)^. Lesions in the cerebellar hemispheres are more likely to produce limb and trunk ataxia, and eye movement disorders reflect vermis dysfunction^(1)^.

The cerebellum is irrigated by branches of the vertebrobasilar system^(3)^. The superior zones of the cerebellar hemispheres and vermis are supplied by the superior cerebellar arteries^(3)^. The inferior zones of the cerebellar hemispheres and vermis are supplied by the posterior inferior cerebellar arteries^(3)^. The intermediate zone of the cerebellum, between the territories of each superior cerebellar artery and posterior inferior cerebellar artery, is irrigated by the anterior inferior cerebellar arteries^(3)^. The cerebellum is drained by three groups of veins^(4)^: superior (galenic), anterior (petrosal), and posterior (tentorial). For adequate functioning, the cerebellum maintains a circuitry that connects it with the spine and supratentorial structures.

The aim of this article is to review various possible causes of ataxia, on the basis of magnetic resonance imaging (MRI) studies obtained from the archives of a tertiary care hospital. The main imaging aspects of the conditions discussed in this article are summarized in Table 1.

**Table 1:** The main imaging aspects of acquired ataxia.

Disease	Etiology	Imaging findings
Tuberculosis	Infectious	Most commonly showing leptomeningitis, with or without pachymeningitis. Tuberculomas of variable size (mean, 2.5 cm) with ring enhancement and liquefied/necrotic centers. Cerebritis, tubercular abscess, and rhombencephalitis in immunocompromised patients.
*Cryptococcus neoformans*	Infectious	Leptomeningeal disease, dilated perivascular spaces, miliary nodules, or granulomatous lesions. Trehalose peak (characteristic of fungal disease) on spectroscopy.
JC virus infection	Infectious	Cerebellar atrophy, with damage and gliosis of the of the pontocerebellar fibers in the pons, producing the “hot cross bun sign”.
Phenytoin intoxication	Toxic	Cerebellar atrophy involving the cerebellar peduncles, together with calvarial thickening.
Stroke and thrombosis	Vascular	Stroke: restricted diffusion in the acute phase with or without a hyperintense signal on T2/FLAIR sequences; cortical enhancement in the subacute phase and atrophy in the chronic phase. Venous thrombosis: lack of venous filling on CT angiography or MR angiography, with adjacent edema or hemorrhage. Enlarged vessel with a markedly hypointense signal on T2*/SWI.
Neuro-Behçet’s disease	Inflammatory	Small lesions with hyperintense signal and enhancement on T2WI/FLAIR, located mostly in the brainstem, and brainstem atrophy without significant cortical atrophy.

## INFECTIOUS DISEASES

### Central nervous system cryptococcosis

*Cryptococcus neoformans* is a saprophytic fungus found in bird feces^(5)^, causing infection in immunocompromised patients^(6)^. Some variants (e.g., *C. neoformans* var. *gattii*) can affect even immunocompetent patients. The agent enters through the respiratory tract and spreads to the central nervous system (CNS) by hematogenous dissemination^(6)^.

**Figure 1:**
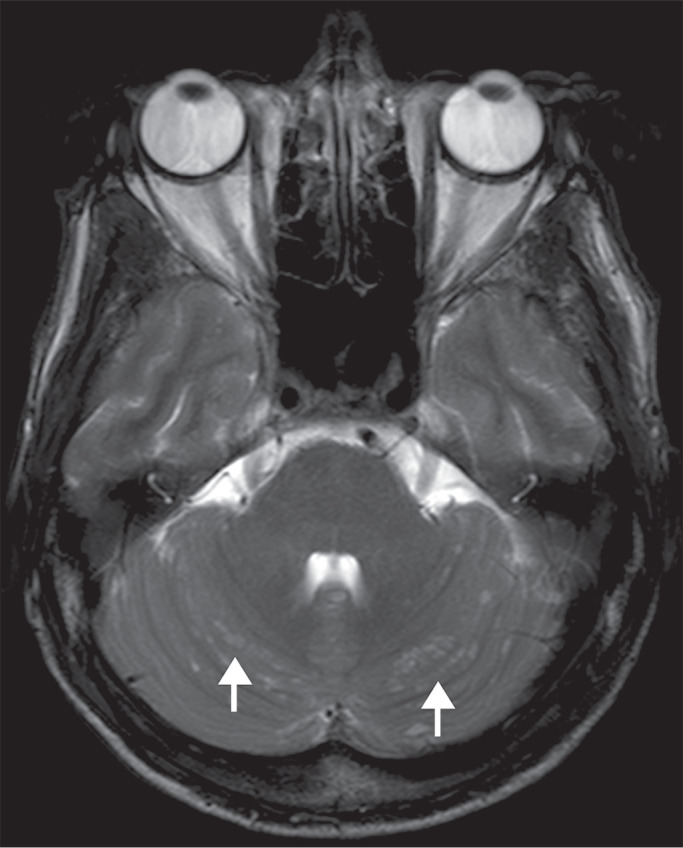
Axial T2-weighted image showing multiple hyperintense ovoid lesions in the cerebellar hemispheres (arrows). Those lesions had demonstrated hypointense signal on T1-weighted images, with punctate enhancement on after contrast administration, without calcifications or bleeding foci and no restricted diffusion (images not shown). The final diagnosis was CNS cryptococcosis.

On MRI, cryptococcal infection may show a variety of features^(5-8)^, including hydrocephalus, leptomeningeal enhancement, dilated perivascular spaces, miliary nodules, plexitis, and pseudotumor (cryptococcoma). Chronic granulomatous reactions are more common in immunocompetent patients^(5-7)^. The MRI pattern seen in the resulting masses is described in Figure 1. Spectroscopy may show a trehalose peak (3.6-3.8 ppm), which is specific for fungal infection.

### CNS tuberculosis

Hematogenous spread from pulmonary infection with *Mycobacterium tuberculosis* can lead to CNS tuberculosis^(9,10)^, which presents as leptomeningitis, with or without pachymeningitis, together with meningeal thickening and enhancement^(10)^. That can be accompanied by hydrocephalus and vasculitis of large cerebral blood vessels^(11)^.

**Figure 2:**
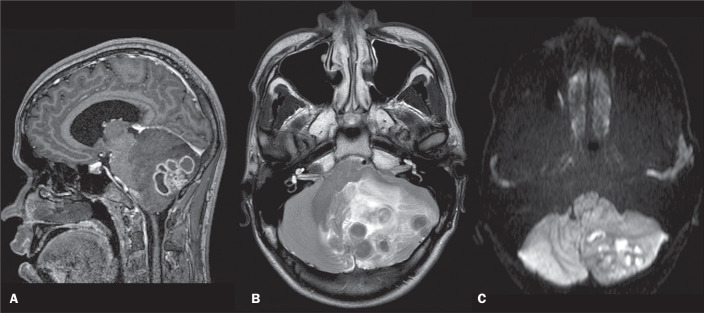
Peripheral ring-enhancing lesions on a contrast-enhanced sagittal T1-weighted image (A). Lesions showing hypointense signal, together with perilesional edema, on a T2-weighted image (B) and marked restricted diffusion on diffusion-weighted imaging (C). The lesions also showed hypointense signal on T1-weighted imaging and on susceptibility-weighted imaging (not shown). The final diagnostic was tuberculosis.

The most common parenchymal manifestation of CNS tuberculosis is the formation of tuberculomas, which are hypointense on T1- and T2-weighted images, often with central liquefaction and nodular or ring-like enhancement^(9,10)^, as depicted in Figure 2. It can present in miliary form, with punctate foci of enhancement usually in immunocompromised patients^(10)^.

Other manifestations of CNS tuberculosis include cerebritis and tubercular abscess, which is an infrequent manifestation (most common in immunocompromised patients) and shares an imaging pattern with pyogenic abscess^(10)^. Tubercular rhombencephalitis, a rare form with a poorer prognosis, is observed in immunocompromised patients^(10)^.

### JC virus infection

Progressive multifocal leukoencephalopathy (PML) is a CNS demyelinating disease caused by the JC virus^(12,13)^. It occurs mainly in patients with severe immunodeficiency, such as those with HIV/AIDS, or individuals treated with monoclonal antibody therapies such as natalizumab^(12)^. Typical PML is characterized by multifocal, bilateral, asymmetrical lesions involving the white matter^(12)^; however, infection of the granular cells of the cerebellar cortex by the JC virus might result in cerebellar atrophy, together with damage and gliosis of the pontocerebellar fibers in the pons, producing the “hot cross bun sign”, characterized by cruciform hyperintense lesion on T2/fluid-attenuated inversion recovery in the pons and rarely depicted in PML^(13)^. The difference among them is believed to be related to a mutant JC virus harboring a small VP1-capsid-protein deletion, which changes the viral tropism^(13)^.

**Figure 3:**
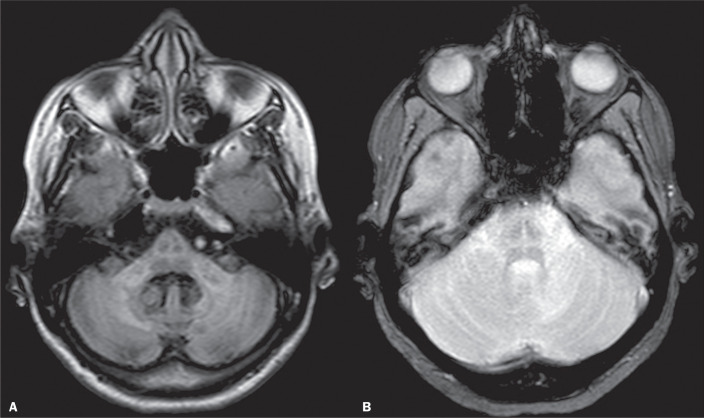
Axial fluid-attenuated inversion recovery sequence (A) and axial T2-weighted image (B) showing a cruciform signal (“hot cross bun sign”) involving the transverse fibers, the median raphe of the pons and the middle cerebellar peduncles, findings that are consistent with JC virus infection.

The typical imaging features of cerebellar JC virus infection are demonstrated in Figure 3^(12)^. The associated lesions may show peripheral restricted diffusion on diffusion-weighted imaging^(12)^. On MRI spectroscopy, an elevation of the choline peak can be observed, as can a reduction in N-acetylaspartate^(12)^.

## TOXIC CONDITIONS

**Figure 4:**
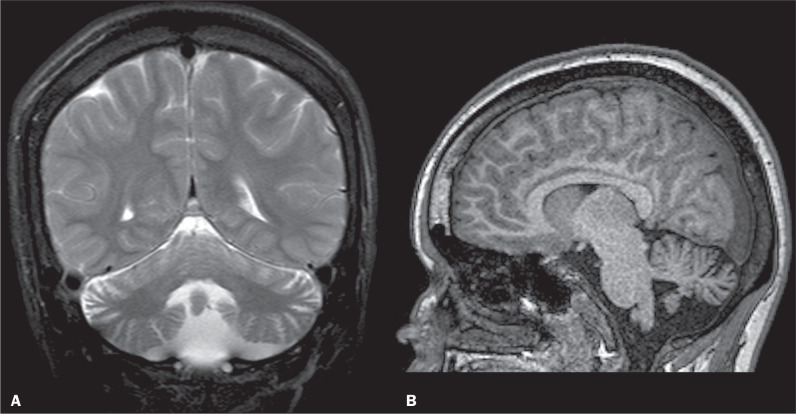
Coronal T2-weighted image (A) showing a reduction in the volume of the cerebellar parenchyma, with evident cerebrospinal fluid among the cerebellar folia, and enlarged fourth ventricle. T1-weighted image (B) showing calvarial thickening. The patient had a history of chronic phenytoin use.

Phenytoin is a hydantoin derivative that functions as aromatic anticonvulsant^(14)^. It is widely accepted that the use of this medication is associated with cerebellar atrophy^(14,15)^, as illustrated in Figure 4. Calvarial thickening is a commonly associated feature.

## VASCULAR DISEASES

**Figure 5:**
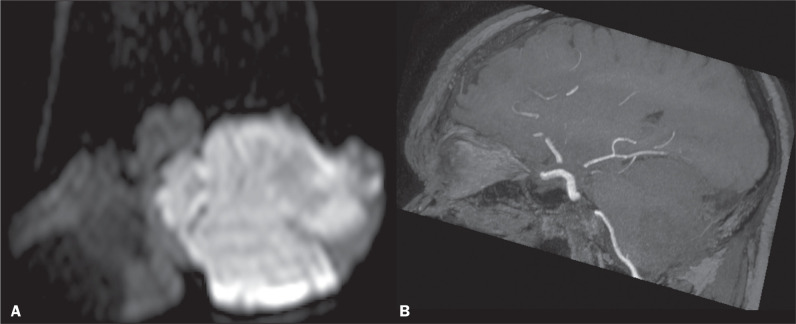
Diffusion-weighted imaging (A) showing restricted diffusion in the left cerebellar hemisphere. Time-of-flight MR angiography (B) demonstrating a lack of flow in the posterior inferior cerebellar artery territory, consistent with cerebellar stroke. A fluid-attenuated inversion recovery sequence showed a hyperintense signal at the same location, with a mass effect compressing the medulla and the inferior cerebellar peduncle (not shown).

Stroke in the cerebellum, lateral medulla or pons, mesencephalon, thalamic nuclei, red nucleus, posterior limb of the internal capsule, and frontal or parietal cortex can manifest as ataxia^(16)^. Cerebellar stroke accounts for approximately 2-3% of all strokes and presents as ataxia, vertigo, diplopia, multidirectional nystagmus, hiccups, dysarthria, nausea, vomiting, hoarseness, dysphonia, or decreased gag reflex. Infarction in the posterior cerebellar artery territory (lateral medullary syndrome, or Wallenberg syndrome) may result in ipsilateral hemiataxia, vertigo, dysarthria, ptosis, or miosis^(16,17)^. Cerebellar ischemia (Figure 5) often occurs in association with brainstem stroke, hypertension and small vessel disease having been implicated as the most common causes^(17)^. Cerebellar hemorrhage accounts for 9-10% of all intracranial hemorrhages.

**Figure 6:**
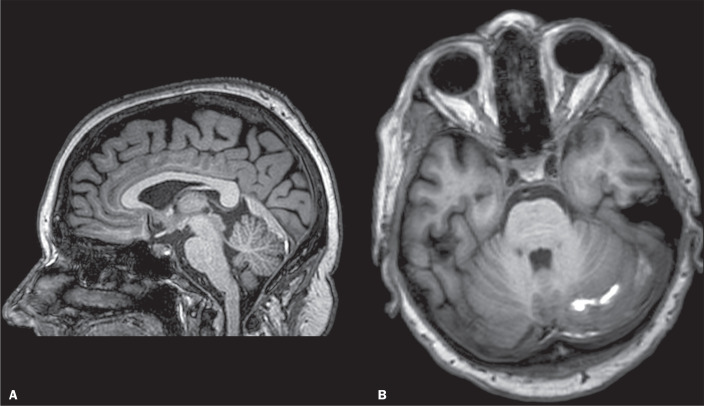
Sagittal T1-weighted image (A) showing a thrombus in the straight sinus and axial T1-weighted image (B) showing a linear hyperintense signal among the cerebellar folia. The final diagnosis was straight sinus thrombosis with subarachnoid hemorrhage and subdural hematoma.

Cerebral venous thrombosis may manifest as cerebellar hemorrhage^(18)^. On unenhanced CT, venous thrombosis presents as a hyperdense sinus or cortical vein^(18,19)^. The MRI findings of venous thrombosis are illustrated in Figure 6. Accurate evaluation of the findings of vascular disease may require a combination of CT and MRI, including diffusion-weighted and susceptibility-weighted imaging or even MR venography^(16)^.

## INFLAMMATORY DISEASES

### Neuro-Behçet’s disease

**Figure 7:**
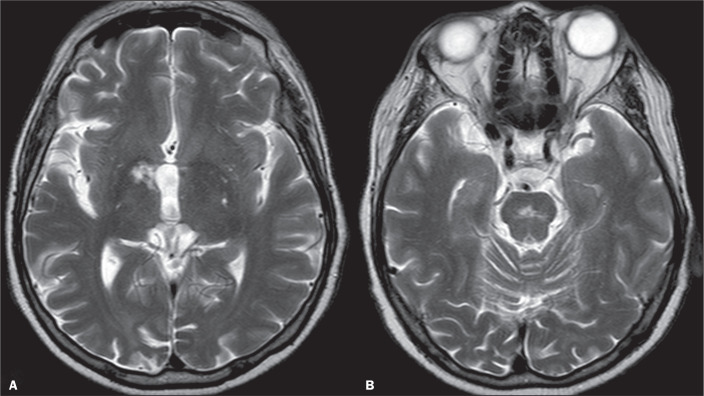
Axial T2-weighted image (A) showing a lesion with a hyperintense signal in the right posterior limb of the internal capsule and in the right ventral thalamus. Axial T2-weighted image (B) showing a hyperintense signal in the midbrain, at the decussation of the superior cerebellar peduncles. On other images (not shown), the lesions do not show enhancement on a contrast-enhanced T1-weighted image and showed markedly hypointense foci on susceptibility-weighted imaging. The patient was in treatment for Behçet’s disease, presenting with ataxia, and the lesions correspond to vascular inflammation related to neuro-Behçet’s disease.

Behçet’s disease is a systemic idiopathic disease characterized by a clinical triad of oral ulcers, genital ulcers, and uveitis, that may be associated with other symptoms as well as arthritis, arthralgia, arterial occlusion or aneurysms and thrombotic events^(20)^. Neuro-Behçet’s disease has two patterns of presentation: parenchymal and nonparenchymal. The former involves the brainstem and cerebral hemispheres, as well as spinal and meningoencephalitis presentations, whereas the latter results in venous sinus thrombosis, intracranial hypertension, arterial occlusion, and aneurysm^(20)^. The site most commonly affected (in 50% of cases) is the brainstem, followed by the white matter, internal capsule, basal ganglia, and thalamus^(20)^. In the brainstem, the pons (tegmentum) and the midbrain are frequently involved^(20)^. Neuro-Behçet’s disease has a variety of MRI findings^(20,21)^, some of which are illustrated in Figure 7.

## CONCLUSION

Ataxia is a syndrome that comprises multiple differential diagnoses and heterogeneous etiologies. Complete anamnesis and detailed clinical inspection are needed in order to establish the time at onset, as well as to identify the signs and symptoms. Investigation of familial disorders and laboratory tests are of paramount importance for conducting an appropriate imaging investigation and interpretation, which are in turn fundamental for obtaining an accurate diagnosis. In this context, radiological interpretation is crucial to enable our clinical colleagues to provide the best available care for each patient.
